# A CSGNN model-based method for essential protein identification

**DOI:** 10.3389/fbinf.2026.1731178

**Published:** 2026-03-16

**Authors:** Zixuan Li, Zhiguo Yu, Peng Li

**Affiliations:** School of Informatics, Hunan University of Chinese Medicine, Changsha, Hunan, China

**Keywords:** dynamic network, dynamic thresholding, essential protein prediction, graph attention network, similarity score

## Abstract

Identification of essential proteins is fundamental for understanding cellular processes and disease mechanisms. However, many existing computational methods do not adequately model dynamic expression activity and often underutilize global network context, which limits prediction accuracy. To address these issues, we propose a Correlation-guided Subgraph Graph Neural Network (CSGNN) for essential protein identification by integrating correlation-guided graph construction with attention-based representation learning. First, we derive an activity-aware expression matrix from periodic gene expression patterns, and we construct a weighted protein network by computing Pearson correlation coefficients between gene pairs. This correlation-guided network further defines first-order and second-order neighborhoods, which provide multi-scale subgraph contexts for each protein. Next, we employ a two-layer attention-based graph convolution to learn node embeddings by aggregating information within these correlation-defined neighborhoods. Finally, we form an interaction-aware node representation by integrating each protein embedding with its neighborhood context, and we use a lightweight multilayer perceptron to output an essentiality probability for each protein. Proteins are then ranked by the predicted scores to identify essential candidates. Experiments on yeast and *E. coli* datasets demonstrate that CSGNN consistently outperforms traditional baselines, indicating improved accuracy and robustness for essential protein identification.

## 1 Introduction

Essential proteins represent a class of key proteins that are indispensable for maintaining cellular viability and normal biological functions. In biological systems, disruption of such proteins, through deletion, inhibition, or functional impairment, often leads to severe growth defects or loss of viability, particularly in microorganisms, indicating their fundamental role in sustaining life processes (Pan and Finkel, 2017). As a result, essential proteins are closely associated with the core proteome that supports basic cellular survival across conditions.

The identification of essential proteins is therefore a core task in life science research and practical applications, playing an important role in understanding biological processes, disease mechanisms, drug development, and targeted therapy. Proteins in living organisms participate in a wide range of functions, including structural support, signal transduction, metabolic regulation, and cellular maintenance (Jones and Thornton, 1996). Identifying those proteins that are essential within complex biological networks enables researchers to gain deeper insights into signaling pathways, metabolic systems, and gene regulatory mechanisms, thereby revealing fundamental principles underlying cellular organization and function.

In the medical domain, essential protein identification provides a foundation for exploring disease mechanisms, discovering pathogenic factors, and developing therapeutic targets (Pan and Finkel, 2017). For example, in cancer and neurodegenerative diseases, identifying essential proteins offers valuable guidance for the design of targeted drugs, improving the precision and effectiveness of diagnosis and treatment (Walkinshaw, 1992). In biotechnology, screening essential proteins is also beneficial for optimizing industrial fermentation processes and developing novel enzyme preparations (Savini et al., 2008). Therefore, essential protein identification constitutes a crucial step in advancing life sciences, both in basic research and in translational and applied studies.

## 2 Related work

Essential proteins are the direct products of gene expression. They play central roles in key cellular processes such as growth, metabolism, and signal transduction (Liu et al., 2024). Accurate identification of essential proteins helps us understand the relationship between genes and phenotypes. It also provides important clues for disease mechanism analysis and drug target discovery.

Traditional experimental methods can validate protein functions with relatively high accuracy. Gene editing (Pace et al., 2024) and protein assembly techniques (Goh et al., 2024) support functional analysis. Gene knockout (Shor and Schneidman-Duhovny, 2024) and RNA interference (Ballantyne et al., 2024) allow direct observation of functional changes *in vivo*. However, these methods are expensive, time-consuming, and limited in throughput. They are not suitable for large-scale systematic screening (Wang et al., 2024). With the development of high-throughput technologies and the accumulation of interaction and expression data, computational identification of essential proteins has become an important research direction. A common paradigm is to use protein–protein interaction (PPI) networks as the backbone and integrate multiple types of biological information (Saha et al., 2024).

In the early stage of computational research, most studies focused on the topological structure of PPI networks. Essential protein identification was treated as a key node detection problem. Proteins were ranked using centrality-based scoring strategies. Degree centrality (DC) was widely adopted because it is intuitive and interpretable. 1t became a classic baseline in this field (Hahn and Kern, 2005). Later, some methods introduced edge weights or neighborhood structure information. These strategies aimed to model interaction strength and local cooperative patterns. They also improved robustness to noisy edges and network sparsity. WDC (Tang et al., 2013) and W5N (Li et al., 2017) are representative examples. They extend the centrality framework by incorporating weights or expanded neighborhood information. As a result, key node detection no longer relies only on static topological features.

Beyond centrality-based and weighted scoring methods, machine learning further promoted the shift from rule-based scoring to data-driven discriminative learning (Inzamam-Ul-Hossain and Islam, 2023). These approaches usually combine PPI topological features with biological features such as Gene Ontology (GO) annotations and expression profiles. A classifier is trained to learn the decision boundary between essential and non-essential proteins. This strategy better captures nonlinear relationships among multiple features. For example, Acencio et al. used several network topological features and two types of GO annotations as inputs to a decision tree model (Inzamam-Ul-Hossain and Islam, 2023). This work provided an early supervised learning framework for multi-source feature integration. Later, Zeng et al. built an ensemble classifier based on gradient boosting decision trees to improve feature representation and prediction accuracy (Zeng et al., 2021). Although these methods improved feature utilization and discriminative performance, they still rely heavily on explicit feature construction and selection. When dealing with high-dimensional, heterogeneous, and noisy biological data, their ability for automatic feature extraction and cross-dataset generalization remains limited (Portugal et al., 2018).

With the rapid development of deep learning in bioinformatics, researchers began to explore end-to-end representation learning frameworks. These frameworks reduce dependence on manual feature engineering. They also integrate network structure, dynamic expression, and sequence semantics in a more systematic way (Zhig et al., 2025). Zeng et al. proposed the DeepEP model. It uses node2vec to learn topological representations from PPI networks. It also applies a convolutional neural network to extract features from gene expression profiles. The two representations are fused to predict essential proteins. This design reflects cooperative modeling of topology and expression features (Ali et al., 2024). To address biological dynamics, Lu et al. proposed the AG-GATCN model. It enhances temporal convolutional networks with attention and gating mechanisms to model dynamic expression patterns. It also uses a graph attention network to extract structural features from PPI networks. This design better captures state-dependent interaction patterns and improves prediction robustness (Yang et al., 2023). Recent studies further incorporate higher-order structural information. For example, Tian et al. proposed the HCNS model. It constructs a hypergraph based on weighted PPI networks and protein complex information. It also integrates sequence features into representation learning. This model captures cooperative structures at the complex level and sequence semantic signals at the same time. It shows strong performance in accuracy and robustness (Tian et al., 2025). Current deep learning models have moved beyond shallow topology-based paradigms. They now adopt multi-scale and multi-modal biological modeling strategies. These models emphasize functional module cooperation, dynamic regulatory context, and sequence-level semantics. As a result, they provide a representation that is closer to the biological mechanisms of essential protein formation and cellular survival dependency.

Despite significant progress, essential protein identification still faces several common challenges. First, PPI data are heterogeneous and contain noise and missing interactions. False connections and incomplete interactions can affect network modeling and prediction stability. Models need to suppress noisy edges and avoid blindly propagating incorrect information across the whole network. Second, protein interactions and gene expression are dynamic and context-dependent. Static networks cannot fully reflect essentiality under different cellular states. Models should use expression activity to capture state differences and learn representations within context-consistent neighborhoods. Third, multi-source biological data are highly heterogeneous. Effective integration while maintaining biological interpretability remains difficult. Models should perform selective aggregation under structural constraints to improve controllability and interpretability. Finally, many methods cannot balance global cooperative structures and local interactions. This limitation weakens the modeling of module-level essentiality mechanisms. An effective model should capture both local interaction details and module-level cooperation patterns.

To address these challenges, we propose the Correlation-guided Subgraph Graph Neural Network (CSGNN) for node-level essential protein prediction. The model is built on a weighted PPI network. It introduces correlation information derived from gene expression time series into the structural modeling process. We construct correlation-guided neighborhood subgraphs to capture interaction patterns that are consistent with specific biological states. The model then performs multi-layer graph representation learning on the constructed subgraphs. It gradually integrates first-order and higher-order neighborhood information to generate node embeddings. A feature fusion and classification module outputs the final essentiality prediction. In this unified framework, CSGNN jointly models PPI topology and state information reflected by expression correlations. Essential protein evaluation is performed within a state-consistent network context rather than relying only on static interactions. This design provides a more direct representation basis for modeling cooperative effects of essential proteins within functional modules.

## 3 Methods

**FIGURE 1:**
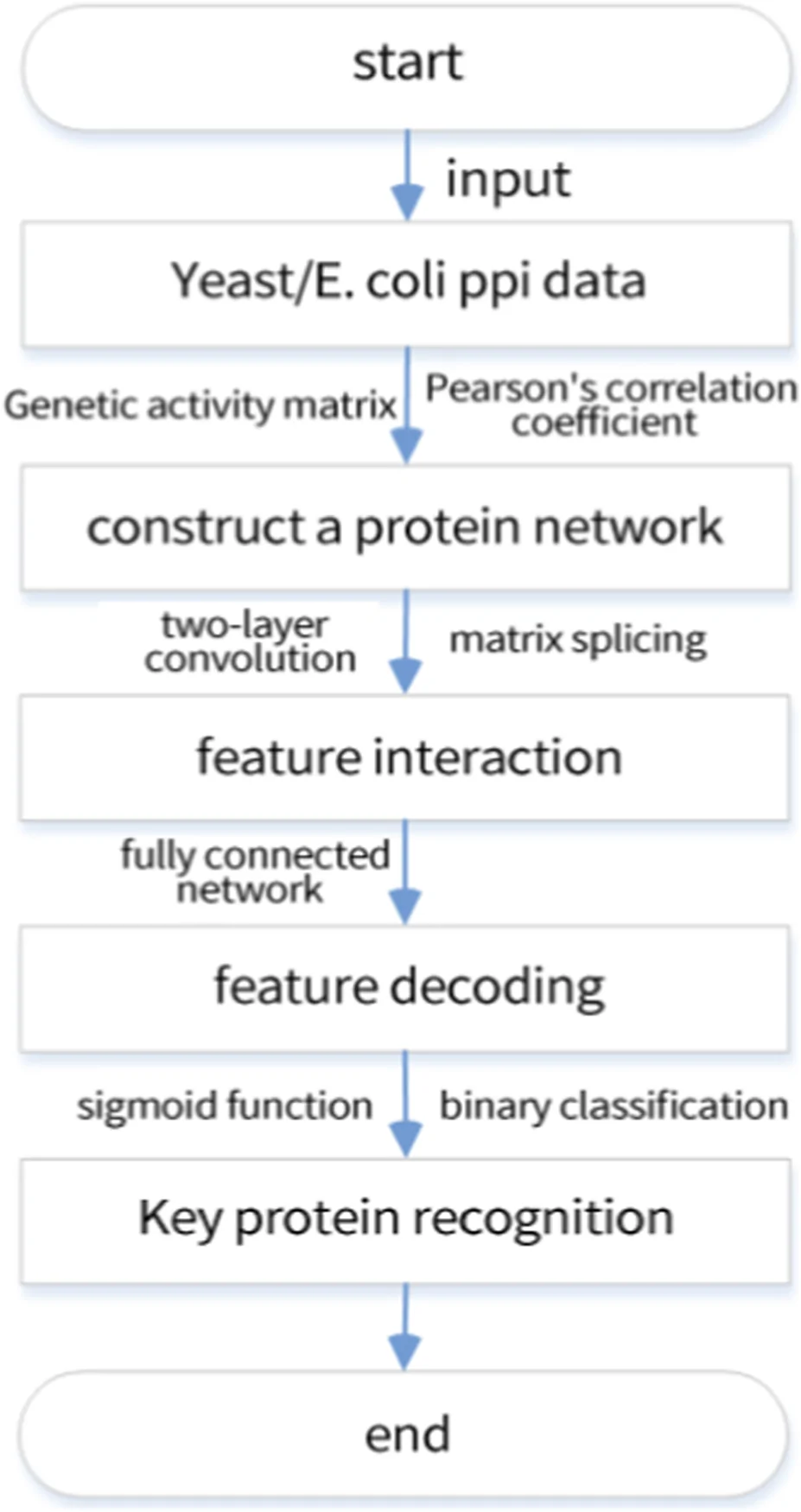
The flowchart for predicting essential proteins.

The overall pipeline of the proposed Correlation-guided Subgraph Graph Neural Network (CSGNN) for node-level essential protein prediction is illustrated in Figure 1. Given a protein–protein interaction (PPI) network and time-series gene expression data, CSGNN first derives activity-aware expression signals to emphasize biologically meaningful temporal variations. Based on these signals, a correlation-guided association structure is constructed to define context-specific neighborhoods (subgraphs) for each target protein, providing a state-consistent local context for representation learning.

CSGNN then performs multi-order neighborhood propagation on the resulting graph structure to capture both direct interactions and broader functional dependencies within local neighborhoods. The learned multi-scale node embeddings are further combined with informative neighborhood evidence in a node-centric manner to form interaction-aware representations. Finally, a lightweight decoder maps these representations to essentiality probabilities, which are converted to binary predictions only during discrete evaluation. Overall, CSGNN integrates temporal activity cues with correlation-guided network topology to learn biologically coherent and discriminative representations for essential protein identification.

### 3.1 Constructing the protein activity feature matrix

Temporal gene expression profiles are shaped by coordinated regulatory programs, including cell-cycle progression and stimulus-responsive transcriptional control (Cubuk et al., 2021). Consequently, observed trajectories typically reflect a mixture of condition-relevant expression changes and background variability. Let M denote the number of measured time points, and let N denote the number of protein nodes in the interaction network.

Using raw expression values ${\left\{x_{i,t}\right\}}_{t=1}^{M}$ directly as node features may introduce low-amplitude variability into neighborhood aggregation, thereby weakening the contrast of condition-relevant temporal patterns and compromising the stability of correlation-based topology construction. We therefore construct an activity-filtered representation by applying a protein-specific dynamic threshold to each temporal profile. This transformation retains expression values that exceed a protein-adaptive baseline and suppresses values that remain within the range of background fluctuation, resulting in node features that better reflect temporally salient regulatory changes.

For protein i , its expression profile is denoted by $d_{i}=\left[x_{i,1},x_{i,2},\ldots ,x_{i,M}\right]$ , where $x_{i,t}$ is the original expression value at time point t . We define the protein-specific dynamic threshold as shown in Equation 1:

$${Threshold}_{i}=\mu_{i}+\frac{2\sigma \left(d_{i}\right)}{1+Var\left(d_{i}\right)}$$ (1)

where $\mu_{i}=\text{mean}\left(d_{i}\right)$ , $\sigma_{i}=\text{std}\left(d_{i}\right)$ , and $\text{Var}\left(d_{i}\right)$ denote the mean, standard deviation, and variance of $d_{i}$ , respectively. The threshold comprises a baseline term $\mu_{i}$ and an adaptive margin controlled by temporal fluctuation. The margin increases with $\sigma_{i}$ , requiring stronger deviations from baseline for profiles with greater dispersion. The factor $1+\text{Var}\left(d_{i}\right)$ moderates the margin under large overall variability, preventing the filtering operation from becoming overly stringent for strongly varying trajectories. Accordingly, the activity-filtered expression is defined as shown in Equation 2:

$$x_{i,t}^{\prime }=\left\{\begin{array}{cc} x_{i,t}, & x_{i,t}\geq {Threshold}_{i}\\ 0, & x_{i,t}<{Threshold}_{i} \end{array}\right.$$ (2)

Finally, we construct the protein activity feature matrix $X^{\prime }\in R^{N\times M}$ , as shown in Equation 3:

$$X^{\prime }=\left[\begin{array}{ccc} x_{1,1}^{\prime } & \cdots & x_{1,M}^{\prime }\\ \vdots & \ddots & \vdots \\ x_{N,1}^{\prime } & \cdots & x_{N,M}^{\prime } \end{array}\right]$$ (3)

Each row corresponds to a protein node and each column corresponds to a time point. The matrix $X^{\prime }$ is used as the node feature input in the subsequent graph neural network.

### 3.2 Constructing protein networks

Cellular functions are rarely executed by isolated proteins; instead, they emerge from coordinated regulatory programs in which groups of proteins exhibit temporally coherent expression patterns (Maier et al., 2020). Such coordinated dynamics reflect shared regulatory control, pathway-level organization, or participation in common functional processes. To capture this structured temporal coordination at the network level, we translate activity trajectories into pairwise associations and construct a protein association graph (Carthew, 2021).

Given the activity-filtered expression matrix $X^{\prime }\in R^{N\times M}$ obtained in Section 2.1, the i -th row vector $x_{i}^{\prime }$ represents the temporal activity profile of protein i across M time points. We quantify the association strength between proteins i and j using the Pearson correlation coefficient computed over their activity trajectories, as shown in Equation 4:

$$Sim\left(i,j\right)=\frac{Cov\left(x_{i}^{\prime },x_{j}^{\prime }\right)}{\sigma \left(x_{i}^{\prime }\right)\sigma \left(x_{j}^{\prime }\right)}$$ (4)

This similarity measure evaluates the consistency of temporal co-variation, thereby capturing coordinated activation and repression patterns under the observed biological condition.

Based on these similarity scores, we construct a static adjacency matrix $A=\left[a_{ij}\right]$ via threshold-based binarization, as shown in Equation 5:

$$a_{ij}=\left\{\begin{array}{cc} 1, & sim\left(i,j\right)>\tau \\ 0, & otherwise \end{array}\right.$$ (5)

where τ controls the sparsity and connectivity of the resulting association network. Since Pearson correlation is symmetric, the adjacency matrix satisfies $a_{ij}=a_{ji}$ .

The threshold τ determines the balance between preserving coordinated temporal structures and suppressing weak or potentially spurious associations. We systematically evaluated model performance across a range of τ values. Predictive performance improves as weak correlations are filtered out, reaches a maximum around τ=0.6 , and declines when the network becomes overly sparse. Smaller thresholds retain excessive low-strength associations and increase noise, whereas larger thresholds fragment the graph and limit the modeling of structured temporal dependencies. Based on this empirical analysis, we set τ=0.6 , achieving the best trade-off between predictive accuracy and topological stability for subsequent graph convolution.

### 3.3 Graph convolution with multi-order, attention-guided aggregation

Essentiality is a systems phenotype that emerges from coordinated cellular organization. Proteins tend to be essential when they execute non-redundant roles in core processes, such as macromolecular complex assembly, metabolic throughput, and regulatory control (Ljubojevic et al., 2021). In expression-derived association graphs, these roles are typically reflected by coherent contextual patterns: proteins within the same functional unit show tightly coupled temporal activity, while weaker associations may result from indirect effects, transient coupling, or measurement variability (Day et al., 2022). As a consequence, essential-protein prediction benefits from an aggregation scheme that emphasizes functionally coherent context and attenuates weak dependencies that can propagate non-specific signals.

To achieve this, CSGNN adopts attention-based message passing and integrates contextual evidence at two structural orders. Let $A=\left[a_{ij}\right]$ be the first-order adjacency matrix defined in Section 2.2, and define a second-order connectivity matrix as shown in Equation 6:

$$A^{\left(2\right)}=A\cdot A$$ (6)

Here ${\left(A^{\left(2\right)}\right)}_{ij}$ indicates the number of length-two paths between proteins i and j ; in this work, we use the condition ${\left(A^{\left(2\right)}\right)}_{ij}>0$ only to identify two-hop reachable neighbors. The first-order structure captures direct co-expression coupling, whereas the two-hop structure captures shared partners and module proximity that arise from modular biological organization.

Let $H^{\left(0\right)}=X^{\prime }\in R^{N\times M}$ denote the input node feature matrix (Section 2.1), and let $h_{i}^{\left(0\right)}\in R^{M}$ be the i -th row vector. We use $\text{ReLU}\left(\cdot \right)$ as the activation function and $\phi \left(\cdot \right)$ as the LeakyReLU used in attention scoring.

#### 3.3.1 First-layer graph convolution

The first graph convolution layer is designed to extract contextual evidence that reflects the local functional environment of each protein. In cellular systems, essential proteins are rarely isolated entities; rather, they are typically embedded in tightly coordinated functional modules, including macromolecular complexes, regulatory circuits, and central metabolic pathways. Within such modules, proteins often exhibit synchronized temporal activity patterns because they are co-regulated, physically coupled, or functionally interdependent (Dudek et al., 2021). Consequently, direct neighbors in the correlation-guided association graph frequently correspond to proteins whose activity trajectories are strongly coordinated with that of the target protein, providing immediate evidence of functional coupling.

However, functional organization in biological networks extends beyond direct interactions. Proteins connected through shared partners may belong to overlapping sub-complexes, participate in adjacent steps of a pathway, or be co-regulated under a common upstream mechanism. Such two-hop connectivity captures a broader layer of module-level organization and may reveal indirect but biologically meaningful dependencies (Guimaraes, 2020). To reflect this hierarchical organization, we explicitly distinguish first-order and second-order structural contexts.

Formally, given the adjacency matrix $A=\left[a_{ij}\right]$ , the first-order neighborhood of node i is defined as $N_{1}\left(i\right)=\left\{\;j|a_{ij}=1\;\right\},$ and the second-order neighborhood is defined as $N_{2}\left(i\right)=\left\{\;j|A_{ij}^{\left(2\right)}>0,j\neq i\;\right\}.$ where $A^{\left(2\right)}=A^{2}$ and the condition ${\left(A^{\left(2\right)}\right)}_{ij}>0$ indicates that proteins i and j are connected by at least one two-hop path. The explicit separation of these two neighborhoods enables the model to treat direct co-expression coupling and broader module proximity as structurally distinct sources of contextual evidence.

Because not all neighbors contribute equally to essentiality inference, we introduce attention-based weighting within each structural order. Even within a coherent functional module, some neighbors exhibit stronger temporal coordination or tighter regulatory dependence with the target protein, whereas others may represent peripheral or condition-specific associations. To capture this heterogeneity, the model first projects node features using a learnable matrix $W^{\left(1\right)}\in R^{d\times M}$ . For each order $m\in \left\{1,2\right\}$ , a compatibility score between node i and its neighbor $j\in N_{m}\left(i\right)$ is computed as shown in Equation 7:

$$e_{ij}^{\left(m\right)}=\phi \left(a^{⊤}\left[W^{\left(1\right)}h_{i}^{\left(0\right)}\|W^{\left(1\right)}h_{j}^{\left(0\right)}\right]\right)$$ (7)

where $q_{m}\in R^{2d}$ is a learnable attention vector specific to structural order m , ∥ denotes concatenation, and $\phi \left(\cdot \right)$ is the LeakyReLU activation. The attention coefficient is then obtained by neighborhood-wise normalization, as shown in Equation 8:

$$a_{ij}^{\left(m\right)}=\frac{\exp\left(e_{ij}^{\left(m\right)}\right)}{\sum_{k\in N_{m}\left(i\right)}\exp\left(e_{ik}^{\left(m\right)}\right)}$$ (8)

These coefficients quantify the relative contribution of each neighbor when updating node i , thereby enabling the model to emphasize neighbors whose representations are more compatible with the target in the projected feature space.

The order-specific aggregated representations are then computed as shown in Equation 9:

$$h_{i,m}^{\left(1\right)}=ReLU\left(\sum_{k\in N_{m}\left(i\right)}a_{ij}^{\left(m\right)}W^{\left(1\right)}h_{j}^{\left(0\right)}\right)$$ (9)

Finally, the first-order and second-order representations are concatenated as shown in Equation 10:

$$h_{i}^{\left(1\right)}=\left[h_{i,1}^{\left(1\right)}\|h_{i,2}^{\left(1\right)}\right]$$ (10)

yielding a multi-scale embedding that simultaneously encodes immediate functional coupling and broader module-level proximity. In biological terms, this layer consolidates coherent local coordination while attenuating weak or inconsistent associations that may otherwise obscure functionally organized patterns relevant to essentiality.

#### 3.3.2 Second-layer graph convolution

While the first layer primarily captures local functional coherence and module-proximal context, essentiality often depends on a protein’s position within a broader and more distributed functional organization (Fuchs et al., 2020). In cellular systems, essential proteins frequently serve as structural scaffolds of complexes, bottlenecks in metabolic pathways, or coordinators of regulatory programs. Their indispensability may therefore arise from coordinated activity patterns spanning multiple interconnected substructures rather than from a single tightly coupled neighborhood (Morris et al., 2022).

After the first layer, the node representations $h^{\left(1\right)}$ already encode refined local consistency and two-hop contextual information. The second layer operates on this enriched representation space and extends information propagation, allowing the model to further consolidate distributed dependencies across overlapping modules or sub-complexes. In this stage, neighbor importance is re-evaluated in light of functionally integrated embeddings rather than raw activity trajectories.

Specifically, a second projection matrix $W^{\left(2\right)}\in R^{d\times 2d}$ is applied to first-layer embeddings. For each structural order $m\in \left\{1,2\right\}$ , attention coefficients $\beta_{ij}^{\left(m\right)}$ are computed analogously to the first layer, but using $h^{\left(1\right)}$ as input. The order-specific updates are defined as shown in Equation 11:

$$h_{i,m}^{\left(2\right)}=ReLU\left(\sum_{j\in N_{m}\left(i\right)}\beta_{ij}^{\left(m\right)}W^{\left(2\right)}h_{j}^{\left(1\right)}\right)$$ (11)

The final node embedding is obtained by concatenation, as shown in Equation 12:

$$h_{i}^{\left(2\right)}=\left[h_{i,1}^{\left(2\right)}\|h_{i,2}^{\left(2\right)}\right]$$ (12)

Through this second aggregation stage, contextual evidence is refined at a higher level of organization. The first layer emphasizes immediate coordination and module adjacency, whereas the second layer integrates more distributed patterns across connected functional regions. The resulting embeddings thus capture essentiality-related signals at complementary biological scales and provide a multi-scale, biologically informed representation for downstream node-level prediction.

### 3.4 Essential protein identification

After two graph convolution layers, each protein is represented by a multi-scale embedding that integrates direct co-expression coupling and two-hop module-proximal context. In cellular systems, essentiality typically emerges from a protein’s participation in coordinated functional organization rather than from isolated molecular activity. Many essential proteins are indispensable because they serve as core components of macromolecular complexes, maintain flux through critical metabolic routes, or coordinate central regulatory programs (Monzel et al., 2023). These roles are inherently contextual: the functional impact of a protein is expressed through stable and consistent relationships with its partners and surrounding functional neighborhood. Accordingly, a reliable essentiality predictor should capture not only node-intrinsic signals but also how coherently a protein aligns with informative contextual proteins in its neighborhood.

To incorporate such neighborhood-consistency evidence while maintaining a strictly node-level prediction target, we construct interaction-aware descriptors between each target protein and its contextual proteins, and aggregate them into a single node-centric representation.

Let $H_{1}^{\left(2\right)}\in R^{N\times d}$ and $H_{2}^{\left(2\right)}\in R^{N\times d}$ denote the second-layer outputs obtained under the first-order and second-order structural contexts, respectively. We concatenate them to obtain the final embedding matrix, as shown in Equation 13:

$$H_{final}=\left[H_{1}^{\left(2\right)}\|H_{2}^{\left(2\right)}\right]\epsilon R^{N\times 2d}$$ (13)

The embedding of protein i is $h_{i}\in R^{2d}$ , i.e., the i -th row of $H_{\text{final}}$ . We define the contextual protein set for i as $C\left(i\right)=N_{1}\left(i\right)\cup N_{2}\left(i\right)$ , which includes proteins connected to i either directly or through two-hop reachability in the correlation-guided association graph. This contextual set provides a structured approximation of the functional neighborhood in which the essentiality of protein i is manifested.

For each contextual protein $j\in C\left(i\right)$ , we construct an interaction descriptor that summarizes complementary aspects of representation compatibility between the target and its context. Specifically, element-wise addition captures shared trends in embedding magnitude and direction, as shown in Equation 14:

$$h_{add}^{\left(i,j\right)}=h_{i}+h_{j}\epsilon R^{2d}$$ (14)

while element-wise multiplication emphasizes feature-wise agreement by highlighting dimensions that are simultaneously strong in both proteins, as shown in Equation 15:

$$h_{prod}^{\left(i,j\right)}=h_{i}⨀h_{j}$$ (15)

In biological terms, this feature-wise agreement is consistent with the fact that essentiality-related evidence often concentrates on coherent functional signals shared by proteins within tightly coordinated modules. We further include ordered concatenation, as shown in Equation 16:

$$h_{cat}^{\left(i,j\right)}=\left[h_{i}\|h_{j}\right]\epsilon R^{4d}$$ (16)

which preserves the target and context embeddings without forcing early mixing. Although the association graph is undirected, the descriptor is constructed in a target–context manner (centered at protein i ) to characterize how well each neighbor j matches the functional context of i , rather than to impose directionality on edges.

We then concatenate the interaction terms to form the pair-level compatibility descriptor, as shown in Equation 17:

$$h_{pair}^{\left(i,j\right)}=\left[h_{add}^{\left(i,j\right)}\left\|h_{prod}^{\left(i,j\right)}\right\|h_{cat}^{\left(i,j\right)}\right]\epsilon R^{8d}$$ (17)

Since the prediction target remains protein i , we aggregate these pair descriptors across $C\left(i\right)$ into a single interaction-aware representation, as shown in Equation 18:

$${\bar{h}}_{i}=\sum_{C\left(i\right)}w_{ij}h_{pair}^{\left(i,j\right)}$$ (18)

The weights $\omega_{ij}$ are derived from the learned attention coefficients in the second graph convolution layer and then re-normalized over the union context set $C\left(i\right)$ . This design ensures that neighbors exhibiting stronger functional consistency with protein i contribute more strongly to the aggregated descriptor, aligning the final decision with coherent module-level evidence rather than with weak or noisy associations. Importantly, although interaction descriptors are constructed at the protein-pair level, the aggregation step yields exactly one representation ${\bar{h}}_{i}$ per node, and the prediction therefore remains strictly node-level.

Finally, we map ${\bar{h}}_{i}$ to an essentiality probability using a two-layer decoder, as shown in Equations 19, 20:

$$z=ReLU\left(W_{1}{\bar{h}}_{i}+b_{1}\right)$$ (19)

$${\hat{y}}_{i}=Sigmoid\left(W_{2}z_{i}+b_{2}\right)$$ (20)

where $W_{1}\in R^{d_{h}\times 8d}$ , $b_{1}\in R^{d_{h}}$ , $W_{2}\in R^{1\times d_{h}}$ , $b_{2}\in R$ , and $d_{h}$ is the hidden dimension. For discrete evaluation, we produce hard labels using a decision threshold δ , as shown in Equation 21:

$${\tilde{y}}_{i}=\left\{\begin{array}{cc} 1, & {\hat{y}}_{i}\geq \delta \\ 0, & {\hat{y}}_{i}<\delta \end{array}\right.$$ (21)

In this study, we use δ=0.5 as the default decision threshold. Since ${\hat{y}}_{i}$ is produced by a Sigmoid function, δ=0.5 corresponds to the neutral decision boundary where the model assigns equal posterior preference to the essential and non-essential classes.

## 4 Experimental data

To evaluate the effectiveness of the proposed CSGNN framework across distinct biological contexts, we conducted experiments on two well-studied model organisms: *Saccharomyces cerevisiae* (yeast) and *E. coli*. Yeast is a canonical eukaryotic system with extensively curated protein–protein interaction (PPI) resources and essentiality annotations, and it has been widely used as a benchmark for essential protein identification. In contrast, *Escherichia coli* is a representative prokaryotic organism with a relatively compact regulatory architecture. Using both organisms allows us to examine the behavior of the proposed method under different network sizes, densities, and biological mechanisms.

The PPI networks of yeast and *E. coli* were obtained from the DIP database (Xenarios et al., 2000). During preprocessing, duplicate interactions and self-interactions were removed to ensure structural consistency. After filtering, the yeast PPI network contains 5,093 proteins and 24,743 interactions, while the *E. coli* network contains 2,727 proteins and 11,803 interactions.

Time-series gene expression data were collected from the Gene Expression Omnibus (Clough and Barrett, 2016). For yeast, dataset GSE3431 contains 6,777 gene products measured at 36 time points, of which 4,858 genes were mapped to proteins in the yeast PPI network. For *E. coli*, dataset GSE3905 contains 7,312 gene products measured at 8 time points. These temporal profiles provide the basis for constructing correlation-guided interaction graphs.

Essential protein annotations were compiled from curated biological databases. For yeast, essentiality information was integrated from MIPS (Pagel et al., 2005), SGD (Dwight et al., 2002), DEG (Zhang and Lin, 2009), and SGDP (Giaever and Nislow, 2014). After mapping to the PPI network, 1,167 essential proteins were retained in the yeast interaction network. For *E. coli*, 254 essential proteins were identified within the constructed PPI network.

In our learning setting, each protein corresponds to a node in a fixed organism-specific network, and the task is to predict a binary essentiality label for each node. The correlation-guided graph is constructed using the available expression profiles and the organism-level interaction network, and model training is performed as node-level supervised learning on this shared graph. We adopt a stratified random split over nodes, with 70%/10%/20% for training/validation/test, while preserving the ratio of essential to non-essential proteins. Only training labels are used to optimize model parameters, and the validation set is used for model selection. Test labels are held out for final evaluation. This design matches a common biological use case: essentiality is experimentally known for only a subset of proteins, and the goal is to infer essential proteins for the remaining uncharacterized proteins within the same organism-level interaction context.

## 5 Simulation experiments

### 5.1 Evaluation indicators

In this study, multiple evaluation metrics are adopted to comprehensively assess the performance of the proposed model on the essential protein prediction task, including Top-N accuracy, area under the ROC curve (AUC), Recall (REC), Matthews correlation coefficient (MCC), F1 score, and Positive Predictive Value (PPV).

Top-N accuracy measures the number of true essential proteins correctly identified among the top N proteins ranked by their predicted essentiality scores, reflecting the model’s ability to prioritize the most critical candidate proteins. This metric is particularly relevant for biological screening scenarios, where only a limited number of top-ranked proteins can be experimentally validated.

The AUC quantifies the area under the receiver operating characteristic (ROC) curve and evaluates the overall discriminative capability of the model across different decision thresholds. A higher AUC value indicates stronger robustness and generalization in distinguishing essential proteins from non-essential ones.

Recall (REC) measures the proportion of true essential proteins that are correctly identified by the model, reflecting its sensitivity in detecting essential proteins. Matthews correlation coefficient (MCC) is a balanced metric that jointly considers true positives, false positives, true negatives, and false negatives, making it particularly suitable for datasets with class imbalance. The F1 score represents the harmonic mean of precision and recall, capturing the trade-off between prediction accuracy and coverage. PPV indicates the proportion of proteins predicted as essential that are indeed essential, reflecting the reliability of the prediction results.

Together, these metrics provide a comprehensive and quantitative evaluation of the model’s performance from multiple perspectives, enabling a thorough assessment of its effectiveness in essential protein identification, as shown in Equations 22, 23:

$$MCC=\frac{TP\times TN-FP\times FN}{\sqrt{\left(TP+FP\right)\left(TP+FN\right)\left(TN+FP\right)\left(TN+FN\right)}}$$ (22)

$$PPV=\frac{TP}{TP+FP}$$ (23)

### 5.2 Experimental environment and parameter settings

In this experiment, we performed model training based on the PyTorch framework using Nvidia GeForce RTX 3090 GPUs and the CUDA 12.1 environment, which ensured the efficiency of the training process. To optimize the training process of the model, we set the learning rate to $1\times 10^{-4}$ , the batch size to 64, and used the AdamW optimizer combined with the weight decay coefficient $1\times 10^{-4}$ to enhance the generalization ability of the model. To dynamically adjust the learning rate, we introduced the ReduceLROnPlateau scheduler, which reduces the learning rate to 0.5 times of the original one when the validation set performance (AUROC) is not improved for 3 consecutive cycles. The training process was carried out for 30 cycles and all training data were normalized and combined with a randomized data loading strategy (shuffle = True) to enhance data diversity. The model performance is monitored in real-time via TensorBoard, which records a number of evaluation metrics such as AUC, F1 score, and Top-100 to ensure comprehensive tracking and analysis of the model performance.

### 5.3 Experimental results and analysis

#### 5.3.1 Top sort analysis

**FIGURE 2:**
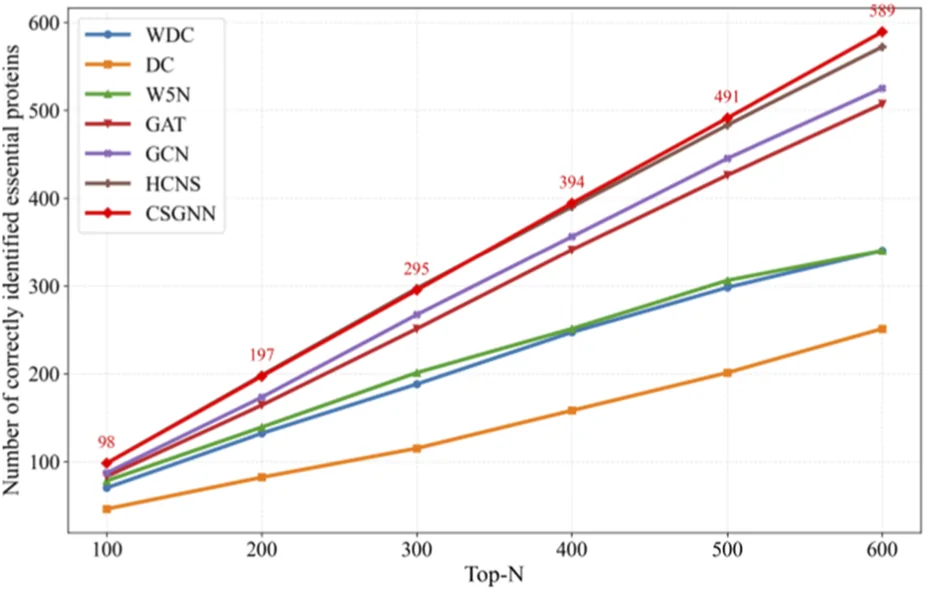
Analysis of the number of top orderings for different prediction methods (yeast data).

**FIGURE 3:**
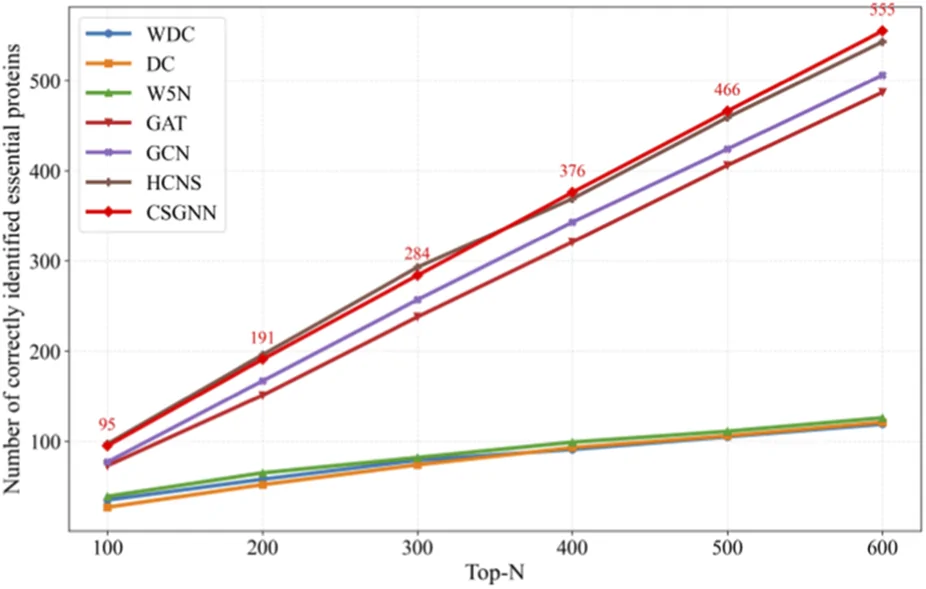
Analysis of the number of top orderings for different prediction methods (*Escherichia coli* data) A similar performance hierarchy is observed on the *Escherichia coli* dataset. The topology-based baselines remain the weakest, identifying only 35, 27, and 39 essential proteins at Top-100 for WDC, DC, and W5N, respectively. Supervised GNN baselines again produce a substantial improvement: GAT achieves 73, 151, 238, 321, 406, and 487, and GCN achieves 77, 167, 257, 343, 424, and 506 from Top-100 to Top-600. At the highest tier, HCNS obtains 97, 196, 293, 369, 459, and 543, while CSGNN achieves 95, 191, 284, 376, 466, and 555. Although HCNS performs slightly better in the smallest Top ranges, CSGNN gradually surpasses it beyond Top-300 and ultimately yields a +12 advantage at Top-600 (555 vs. 543). This trend indicates that CSGNN maintains more stable ranking behavior as the evaluation shifts from the very top candidates to a broader decision range.

To further evaluate the ranking capability of CSGNN, we conducted a Top-N analysis on the yeast and *E. coli* datasets. For each model, we report the number of correctly identified essential proteins within the Top-100 to Top-600 ranked candidates. In addition to three representative topology-based methods, WDC (Tang et al., 2013), DC (Hahn and Kern, 2005), and W5N (Li et al., 2017), we include two standard supervised GNN baselines, namely, a vanilla two-layer GCN and a two-layer multi-head GAT implemented under the same data splits and training protocol. We further compare against HCNS, a recent state-of-the-art framework integrating hypergraph convolution with deep sequence modeling. The results are illustrated in Figures 2, 3.

On the yeast dataset, traditional topology-driven methods show limited ranking capability. At Top-100, WDC, DC, and W5N identify 70, 46, and 78 essential proteins, respectively. Although their performance increases gradually with larger Top-N ranges, their overall growth remains considerably below that of learning-based approaches. Introducing supervised message passing substantially improves ranking quality. The two-layer GAT achieves 83, 164, 251, 341, 426, and 507 correct identifications from Top-100 to Top-600, while the two-layer GCN further improves these numbers to 87, 173, 267, 356, 445, and 525. The consistent advantage of GCN over GAT across all Top ranges suggests that, in yeast PPI networks, stable normalized neighborhood aggregation provides a more robust ranking signal than attention-based reweighting under noisy interaction edges and class imbalance.

Building upon stronger structural modeling, HCNS and CSGNN further enhance the ranking performance. HCNS reaches 98, 198, 297, 390, 483, and 572 across Top-100 to Top-600, benefiting from the integration of hypergraph structure and deep sequence features. CSGNN achieves 98, 197, 295, 394, 491, and 589, remaining competitive in the smallest Top ranges while showing clearer advantages as the ranking scope expands. In particular, at Top-600 CSGNN identifies 589 essential proteins compared with 572 for HCNS, indicating stronger global ranking stability when a broader set of candidate proteins is considered.

Taken together, the results across both datasets reveal a clear methodological progression. Traditional centrality-based approaches rely solely on static topological indicators and therefore provide limited ranking discrimination. Standard GCN and GAT introduce supervised neighborhood aggregation and substantially improve the ordering of candidate proteins, yet remain constrained by shallow message passing on a fixed graph. HCNS further incorporates hypergraph modeling and sequence-level representations, enhancing structural and functional feature integration. CSGNN extends this progression by constructing a correlation-guided dynamic interaction network and propagating information over multi-order neighborhoods, enabling interaction-aware representations that better align with the biological characteristics of essential proteins. This design leads to consistently improved global ranking quality, particularly in larger Top-N regimes where ordering stability becomes critical.

#### 5.3.2 Comprehensive multi-metric performance evaluation

To comprehensively evaluate the classification performance of CSGNN, we conduct a multi-metric assessment on the yeast and *E. coli* datasets. The evaluation metrics include AUC, recall (REC), F1 score, Matthews correlation coefficient (MCC), and positive predictive value (PPV). These metrics measure discrimination ability, coverage of true essential proteins, precision–recall balance, and robustness under class imbalance.

Under the supervised node classification framework, each protein in the PPI network is treated as a node-level sample. Proteins annotated as essential in curated biological databases are labeled as positive samples. All remaining proteins are labeled as negative samples. A stratified random partition strategy is adopted. The data are divided into training, validation, and test sets at a ratio of 70%, 10%, and 20%. The proportion of essential and non-essential proteins is preserved in each subset. Model parameters are optimized using only the training set. The validation set is used for hyperparameter tuning. The test set is strictly held out for final evaluation.

The results on the yeast dataset are shown in Table 1. The three topology-based baselines (WDC, DC, and W5N) show limited performance. Their AUC values range from 0.6705 to 0.7152. Their MCC values remain relatively low. These results indicate that static centrality measures or shallow fusion strategies are insufficient for capturing complex interaction patterns in PPI networks.

**TABLE 1:** Multi - performance evaluation analysis (yeast data).

Dataset	Method	AUC	REC	MCC	F1	PPV
Yeast	WDC	0.6893	0.4576	0.2967	0.4578	0.4580
	DC	0.6705	0.4002	0.2219	0.4002	0.4002
	W5N	0.7152	0.4747	0.3186	0.4747	0.4747
	GAT	0.7832	0.7212	0.4872	0.6122	0.6035
	GCN	0.8032	0.7412	0.5232	0.6382	0.6286
	HCNS	0.8772	0.8538	0.6326	0.7936	0.7735
	CSGNN	0.8993	0.8682	0.6284	0.8189	0.7966

Supervised graph learning substantially improves performance. The two-layer GAT achieves an AUC of 0.7832 and an MCC of 0.4872. The two-layer GCN further improves these values to 0.8032 and 0.5232. GCN consistently outperforms GAT across all metrics. This suggests that normalized neighborhood aggregation provides a stable ranking signal under noisy interactions and moderate class imbalance.

HCNS further enhances structural modeling by integrating hypergraph convolution with deep sequence encoders. It achieves an AUC of 0.8772 and an MCC of 0.6326. CSGNN achieves an AUC of 0.8993, a recall of 0.8682, an F1 score of 0.8189, and a PPV of 0.7966. Its MCC is 0.6284, which is comparable to HCNS. However, CSGNN shows higher AUC, recall, and F1. These results indicate stronger global discrimination and a more balanced precision–recall trade-off.

The results on the *E. coli* dataset are presented in Table 2. A similar hierarchy is observed. The topology-based methods achieve AUC values between 0.6837 and 0.7243. Their recall values remain low. The two-layer GAT reaches an AUC of 0.7531 and an MCC of 0.4374. The two-layer GCN improves these to 0.7721 and 0.4574. GCN again performs slightly better than GAT.

**TABLE 2:** Multi-performance evaluation analysis (*Escherichia coli* data).

Dataset	Method	AUC	REC	MCC	F1	PPV
*E. coli*	WDC	0.6837	0.2323	0.1534	0.2323	0.2322
	DC	0.6849	0.2559	0.1795	0.2559	0.2559
	W5N	0.7243	0.2913	0.2186	0.2913	0.2913
	GAT	0.7531	0.6949	0.4374	0.6328	0.6214
	GCN	0.7721	0.7032	0.4574	0.6609	0.6532
	HCNS	0.8182	0.7430	0.5123	0.7248	0.7339
	CSGNN	0.7973	0.7505	0.4713	0.7394	0.7487

HCNS achieves the highest AUC (0.8182) and MCC (0.5123) on this dataset. CSGNN achieves an AUC of 0.7973, a recall of 0.7505, an F1 score of 0.7394, and a PPV of 0.7487. Although its AUC and MCC are slightly lower than those of HCNS, CSGNN obtains higher recall and competitive F1 performance. This indicates that CSGNN identifies a larger proportion of true essential proteins while maintaining stable predictive precision under stronger class imbalance.

Across both datasets, a clear methodological progression can be observed. Traditional centrality-based methods rely on static topology. Their discrimination capacity is limited. Standard GCN and GAT introduce supervised neighborhood aggregation and significantly improve classification performance. However, they are constrained by shallow message passing on a fixed interaction graph. HCNS integrates hypergraph structure and sequence-level representations, which strengthens structural and functional modeling. CSGNN further extends this progression. It constructs correlation-driven interaction graphs and performs multi-order propagation. This design captures dynamic coordination patterns associated with essentiality. As a result, CSGNN achieves strong recall and balanced multi-metric performance across species.

### 5.4 Ablation experiment

**FIGURE 4:**
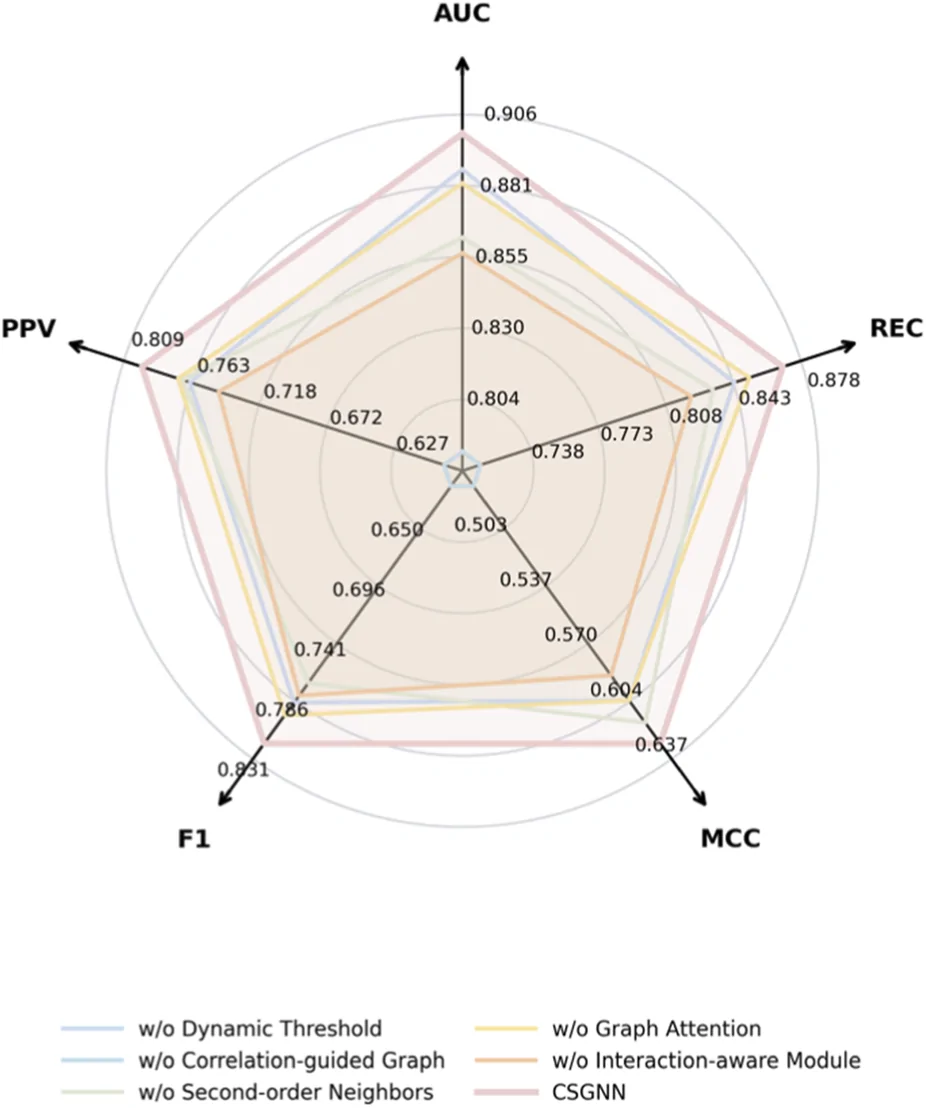
Ablation study on the yeast dataset across five evaluation metrics.

To further investigate the contribution of each architectural component in CSGNN, we conduct a systematic ablation study on the yeast dataset. The evaluation includes AUC, recall (REC), MCC, F1 score, and PPV. The full CSGNN model achieves 0.8993 AUC, 0.8682 recall, 0.6284 MCC, 0.8189 F1, and 0.7966 PPV. Figure 4 illustrates the radar comparison among the full model and its simplified variants.

We first examine the effect of removing the dynamic thresholding mechanism. Without dynamic thresholding, AUC decreases to 0.8865 and recall drops to 0.8432. The decline is consistent across all metrics. This indicates that adaptive correlation filtering improves the quality of the constructed interaction graph. Static thresholds fail to fully capture condition-dependent expression relationships, which weakens the global discrimination ability of the model.

The most significant performance degradation occurs when the correlation-guided graph construction is removed. In this case, AUC drops to 0.7857 and MCC decreases to 0.4783. F1 and PPV are also substantially reduced. This confirms that the correlation-driven graph is the structural foundation of CSGNN. Without this module, the model essentially degenerates into a conventional graph learning framework on a static topology. The results demonstrate that correlation-guided graph construction contributes the largest share of performance gain.

When second-order neighborhood propagation is removed, the performance shows a moderate but consistent decline. AUC decreases to 0.8621 and recall to 0.8321. This suggests that higher-order structural information enhances the model’s ability to capture functional coordination patterns beyond immediate neighbors. Essential proteins often participate in coordinated biological processes that span multiple interaction hops. Limiting propagation to first-order neighborhoods reduces the capacity to model these patterns.

Removing the graph attention mechanism also leads to measurable degradation. AUC decreases to 0.8815 and MCC to 0.6032. Although the performance drop is smaller than that observed for graph construction removal, attention-based weighting improves the discrimination of heterogeneous interaction strengths. Uniform aggregation introduces additional noise, especially in densely connected regions of the PPI network.

The interaction-aware representation module also plays an important role. Without this component, AUC decreases to 0.8565 and recall to 0.8209. This module enables context-sensitive feature refinement based on interaction patterns. Its removal weakens the model’s ability to align structural signals with functional indispensability.

Overall, the ablation results reveal a clear hierarchy of component importance. Correlation-guided graph construction provides the dominant structural advantage. Dynamic thresholding and multi-order propagation further enhance topological expressiveness. Attention weighting and interaction-aware modeling refine representation quality. The consistent degradation across all simplified variants confirms that the performance improvement of CSGNN does not arise from a single architectural modification, but from the coordinated integration of dynamic graph construction and multi-level representation learning.

## 6 Conclusion

In this study, a essential protein prediction method based on Correlation-guided Subgraph Graph Neural Network (CSGNN) is proposed considering the advantages of graph neural networks in processing graph data. The experimental results of applying it to yeast and *E. coli* datasets show that the model exhibits high prediction performance in several key indicators, especially in dealing with the dynamic network characteristics, and is able to identify the essential protein s more accurately than the traditional methods. This validates the potential of graph neural networks for applications in complex biological networks. Future research can further advance the progress of essential protein prediction in several directions. The potential of multimodal data fusion can be explored by combining more biological data, such as gene mutations, protein structural information, and cell type-specific gene expression data, to further improve prediction accuracy. In addition, cross-species prediction of essential protein s can be an important direction to improve the generalization ability of the model by using the shared knowledge between species. With the continuous accumulation of large-scale biological data, the potential of model application on different tasks and datasets can also be enhanced in the future through methods such as migration learning. These directions will promote the widespread application of essential protein prediction techniques to aid biological and medical research.

## Data Availability

The source code and datasets supporting the findings of this study are publicly available at: https://github.com/Lzx2294159762/CSGNN.
