# Correction: A CSGNN model-based method for essential protein identification

**DOI:** 10.3389/fbinf.2026.1986024

**Published:** 2026-09-14

**Authors:** Zixuan Li, Zhiguo Yu, Peng Li

**Affiliations:** School of Informatics, Hunan University of Chinese Medicine, Changsha, Hunan, China

**Keywords:** dynamic network, dynamic thresholding, essential protein prediction, graph attention network, similarity score

Authors Zixuan Li and Zhiguo Yu were erroneously assigned as corresponding authors. The correct corresponding author is Peng Li.

The original version of this article has been updated.

